# Artificial Intelligence-Based Depression Detection

**DOI:** 10.3390/s26020748

**Published:** 2026-01-22

**Authors:** Gabor Kiss, Patrik Viktor

**Affiliations:** 1 Institute of Safety Science and Cybersecurity, Obuda University, 1034 Budapest, Hungary; 2 Keleti Károly Faculty of Business and Management, Obuda University, 1034 Budapest, Hungary; viktor.patrik@uni-obuda.hu

**Keywords:** depression, recognition, artificial intelligence, aviation, road transport, safety

## Abstract

Decisions made by pilots and drivers suffering from depression can endanger the lives of hundreds of people, as demonstrated by the tragedies of Germanwings flight 9525 and Air India flight 171. Since the detection of depression is currently based largely on subjective self-reporting, there is an urgent need for fast, objective, and reliable detection methods. In our study, we present an artificial intelligence-based system that combines iris-based identification with the analysis of pupillometric and eye movement biomarkers, enabling the real-time detection of physiological signs of depression before driving or flying. The two-module model was evaluated based on data from 242 participants: the iris identification module operated with an Equal Error Rate of less than 0.5%, while the depression-detecting CNN-LSTM network achieved 89% accuracy and an AUC value of 0.94. Compared to the neutral state, depressed individuals responded to negative news with significantly greater pupil dilation (+27.9% vs. +18.4%), while showing a reduced or minimal response to positive stimuli (−1.3% vs. +6.2%). This was complemented by slower saccadic movement and longer fixation time, which is consistent with the cognitive distortions characteristic of depression. Our results indicate that pupillometric deviations relative to individual baselines can be reliably detected and used with high accuracy for depression screening. The presented system offers a preventive safety solution that could reduce the number of accidents caused by human error related to depression in road and air traffic in the future.

## 1. Introduction

Over the past two decades, significant technological advances have been made to improve road and air transport safety, particularly in the field of sensor-based driver assistance and driver monitoring systems. Advanced Driver Assistance Systems (ADAS), such as adaptive cruise control, lane departure warning and lane keeping systems, blind spot monitoring, and driver fatigue and inattention monitoring solutions, are now widely used in modern vehicles. The primary goal of these systems is to reduce the number of accidents by the early detection of deteriorating driving performance.

The vast majority of current solutions rely on vehicle dynamics data (steering patterns, lane position, acceleration and braking profiles) and simple visual characteristics (eyelid closure, blink rate, gaze direction). These systems are effective at detecting fatigue, drowsiness, or acute inattention and provide warning signals to the driver. However, their operation is essentially reactive: they send an alarm after detecting a dangerous condition, but do not actually prevent the vehicle from being used.

A further limitation is that most of these systems can be partially or completely switched off, bypassed, or ignored. This design philosophy is based on the assumption that drivers are fundamentally rational decision-makers who follow warnings for their own safety and that of others. However, this assumption does not hold true for all mental states.

### 1.1. Depression as a Critical but Under-Addressed Road Safety Risk

Road safety research has traditionally focused on the effects of fatigue, alcohol and drug use, and inattention. In contrast, the role of mental disorders, particularly depression, in the development of traffic risks has received relatively little attention, despite the fact that depression is one of the most common mental illnesses worldwide.

More severe depressive states are characterised by a number of cognitive and affective distortions that can directly affect driving performance. These include narrowed attention, the increased processing of negative information, reduced responses to positive stimuli, slowed decision-making, and impaired impulse control. In extreme cases, depression can lead to self-harming or even suicidal behaviour.

In a transportation setting, this risk endangers more than just the person affected. The decisions of a depressed driver or pilot can have a direct impact on passengers, other road users, and society at large. One of the most well-known tragedies in the history of aviation, the crash of Germanwings Flight 9525, highlighted that mental health—especially depression—is not only a health issue, but also a critical safety factor.

While alcohol and drug use can be checked objectively, quickly, and in a standardised manner before driving, the detection of depression is currently based primarily on self-reporting, clinical interviews, and time-consuming psychological tests. These procedures are not suitable for real-time decision support relevant to transportation safety.

### 1.2. Objective Physiological Correlates of Depression: Pupillometry and Eye Movements

In recent years, a growing number of studies have examined the objective, measurable physiological correlates of depression, with a particular focus on eye movements and pupillary responses. Pupillometry, as a sensory measurement method, allows for the high-resolution recording of pupil diameter and dynamics, typically using camera-based infrared eye-tracking systems.

Numerous studies have shown that pupil reactions are not solely dependent on lighting conditions, but are closely related to the functioning of the autonomic nervous system, emotional processing, and cognitive load. In depressed individuals, increased or prolonged pupil dilation in response to negative emotional stimuli is consistently observed, while the response to positive stimuli is reduced or completely absent.

Pupillometry results are supplemented by observations from the analysis of eye movement patterns. In the context of depression, changes can be observed in the speed and amplitude of saccades, the duration of fixations, and the spatial distribution of gaze. These patterns are consistent with the attentional distortions and information processing characteristics characteristic of depression.

### 1.3. Limitations of Current Research and Gaps in Application

Although pupillometry and eye movement-based depression research has produced scientifically sound results, most existing studies serve primarily clinical, diagnostic, or psychological purposes. Few studies have addressed how these physiological signals could be directly applied in safety-critical systems, particularly in the field of transportation. Most existing approaches treat depression as an isolated phenomenon and do not integrate it into a comprehensive, real-time decision support system. In addition, they often use thresholds based on population averages, ignoring the significant individual variability that exists in both pupil responses and eye movements. This application gap is particularly problematic in the context of traffic safety, where both false positive and false negative decisions can have serious consequences. Therefore, a solution is needed that not only recognises patterns indicative of depression, but also interprets them in relation to the specific individual and their changes over time.

### 1.4. Proposed Theoretical Framework: Depression Detection as Preventive Access Control

This study presents a theoretical model that interprets the detection of depression not as a clinical diagnosis but as a preventive access control mechanism. The approach draws an analogy with alcohol impairment checks: the goal is not to medically assess mental state, but to determine whether there is a risk at that moment that would justify temporarily restricting driving or conducting further tests.

A key element of the system is the integration of iris-based biometric identification. Iris recognition ensures that the measured pupillometry and eye movement data are always assigned to the correct person. This is particularly important in environments where multiple users may use the same vehicle.

### 1.5. Personalised, Longitudinal AI-Based Model and Research Question

The third innovative element of the presented model is the longitudinal, personalised approach. Depression is a dynamic and heterogeneous condition that changes over time and can manifest in different mental and physiological patterns. Accordingly, the system does not make decisions based on absolute thresholds, but rather examines the extent to which the currently measured pupil and eye movement time series deviate from the previously recorded reference states of the individual. To process the measured time series, the study proposes an architecture that combines a convolutional neural network and long-short term memory (CNN-LSTM), which is suitable for extracting spatial features and modelling temporal dynamics. Accordingly, the central question of the research is as follows:

**Is an artificial intelligence model based on a CNN–LSTM architecture using pupillometry and eye movement time series capable of recognising physiological response patterns indicative of depression with sufficient accuracy and reliability for preventive application in safety-critical transportation environments, particularly in road traffic?**

The rest of this study seeks to answer this question by presenting the proposed theoretical system, detailing the methodology, and analysing the results.

**Figure 1:**
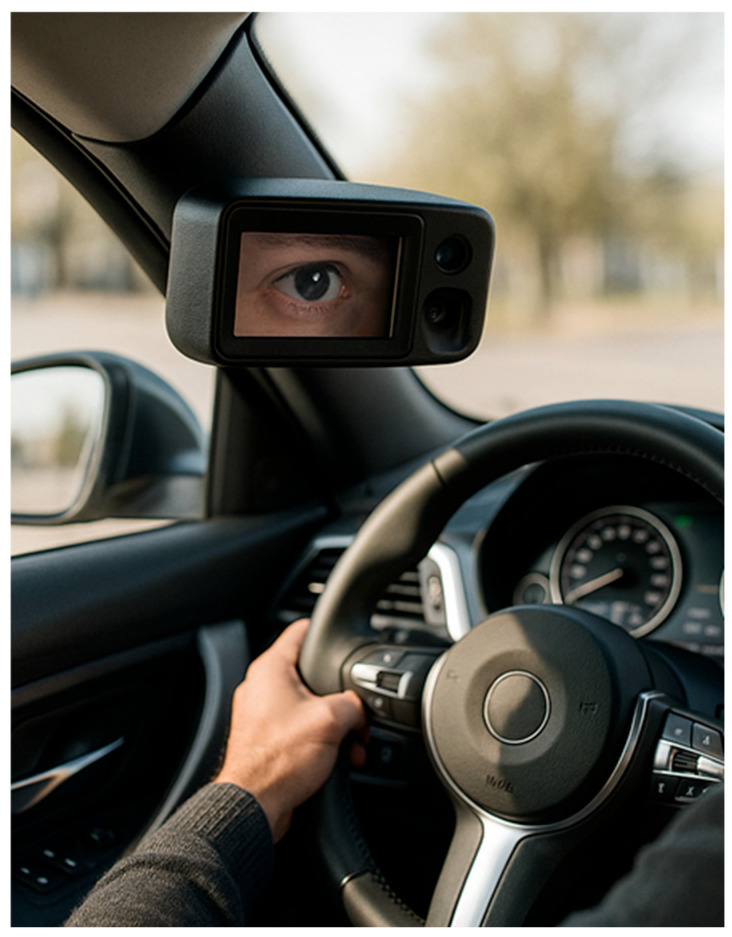
Driver monitoring system/eye-tracking camera in vehicles source [1].

The emphasis is not on the medical assessment of mental state, but on risk prediction and prevention (Figure 1).

## 2. Literature Review

### 2.1. Recognising Depression

Current diagnostic methods for recognising depression typically rely on patients’ subjective self-reports and clinical observations [1], which poses a serious challenge to the accurate identification of depression with mixed symptoms, increasing the risk of both misdiagnosis and missed diagnoses, which can lead to delays in recognising suicidal tendencies [2]. There is currently no reliable and objective biomarker for depression in routine clinical practice, with the result that more than 50% of patients do not receive adequate treatment and an estimated 30% do not improve with currently available medications [3], making the development of a reliable diagnostic tool or system urgent.

Eye movement testing, such as following a doctor’s raised finger, has long been used in medicine in cases of head injury, but it can also help in the diagnosis of certain mental illnesses, such as schizophrenia and autism [4]. This is because the eyes are the only externally visible part of the central nervous system, offering a unique and non-invasive means of examining brain activity and physiological states, and thus of studying the neurobiological mechanisms underlying mental health (Table 1) [4].

Recently, research has also begun to focus on detecting depression based on eye movements [4]. Dynamic eye movement data provides immediate feedback on how a person processes visual information and responds to their environment. Pupil size is closely related to psychological state and reveals the details of a person’s higher cognitive processes. Pupil dilation in this study is not merely a response to light [5], as it plays a very important role in the transmission of information related to uncertainty and surprise during decision-making, thus allowing for the measurement of emotional responses and predictive processing [6].

Depressive illness is associated with cognitive impairment. For example, patients are unable to revise negative beliefs, even when presented with new, positive information. This manifests itself in a reduced response of the pupils to unexpected positive information in individuals with high depressive symptoms. Negative emotional stimuli (e.g., sad faces, negative words) are associated with increased pupil dilation in depressed individuals. These specific, observable eye responses provide physiological evidence for the well-documented cognitive and attentional biases observed in depression [7].

The developed technology can quantify cognitive processes by focusing on attention sharing and emotional states based on gaze direction, eye movement speed, and eye movement trajectory.

Several studies have already investigated the use of iris uniqueness, which has long been used in access control systems, for the detection of various neurological diseases [8,9], but the currently accepted testing method is based on the analysis of the dynamic physiological responses of the eye mentioned above. In 2024, a research group began using artificial intelligence in this field as well [10].

We can see that research on depression detection is already showing significant progress, with some studies even incorporating the use of artificial intelligence.

### 2.2. Iris-Based Identification

**Figure 2:**
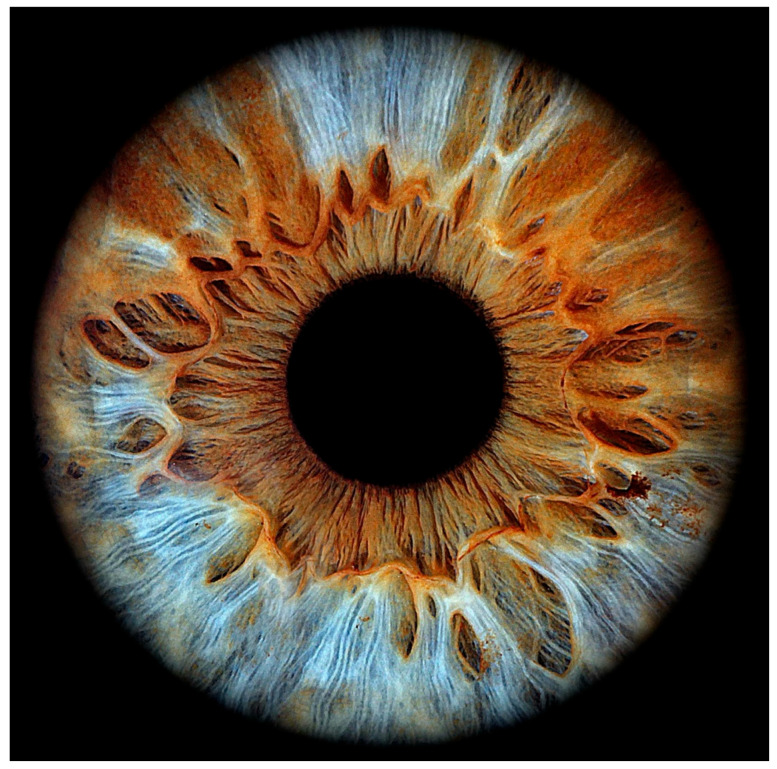
Iris source [1].

Iris-based identification is based on the iris pattern, which is the coloured, ring-shaped part surrounding the pupil [4]. The iris is unique to each individual and extremely detailed. Since it develops randomly before birth, its pattern is different, even in identical twins (Figure 2) [11]. Two additional characteristics make it even more useful for biometric identification. One characteristic is that it remains unchanged throughout a person’s life, with only minor variations occurring with age. The other characteristic is that it is located behind the cornea, making it more protected from damage [12].

Iris scanners are used for identification (Table 2). These are special cameras that can take high-resolution images. Iris recognition algorithms search for the inner (outside the pupil) and outer (inside the limbus) ring boundaries, then determine approximately 240 unique pattern characteristics, which are finally converted into a 512-digit binary identifier that becomes the individual’s iris ID [13]. More advanced systems also use infrared light to reveal details of the iris, regardless of eye colour, as melanin is transparent to infrared light [14].

Since identification is contactless, it is more acceptable to users and is generally not affected by eyeglasses or contact lenses, although patterned contact lenses may interfere with identification [15]. Factors affecting the security of identification are more related to the pupil being too dilated and the iris ring being smaller, thus offering fewer identification points, but this can be eliminated by using a 512-bit biometric identification code, i.e., a sufficiently large unique pattern [16]. If necessary, a 1024-bit or even larger unique identifier can be created by recording even more unique pattern data, which increases security but slightly increases the time required for individual identification. Recently, research has also appeared in the field of iris identification using artificial intelligence for segmentation [17] or for identifying the covered surface of the iris [18].

**Table 2:** Advantages and limitations of iris biometrics source [19].

Properties	Advantages	Barrier
Uniqueness	High security	–
Stability	Does not change for years	–
Infrared transmission	Works even with dark eyes	–
Pupil dilation	–	May impair identification
Contact lenses	–	Patterned lenses interfere

### 2.3. The Current Status and Open Challenges of Pupillometry and Eye Movement-Based Biomarkers of Depression

Over the past decade, objective, physiologically based detection of depression has received increasing attention in the literature, particularly in parallel with the recognition that traditional diagnostic methods rely heavily on subjective self-reports. Pupillometry and eye movement analysis represent a promising avenue of research in this context, as the eye is a directly observable part of the nervous system and provides non-invasive information about cognitive and affective processes [20,21]. Numerous studies have shown that negative emotional stimuli elicit increased or prolonged pupil dilation in depressed individuals, while responses to positive stimuli are reduced or absent [22,23]. These differences are consistent with cognitive–affective models of depression, particularly those involving the excessive processing of negative information and reduced reward sensitivity. One of the main advantages of pupillometry is its ability to track changes in emotional and cognitive load with high temporal resolution, making it suitable for real-time applications [7]. Eye movement characteristics, including saccade speed, fixation duration, and gaze distribution, also show consistent differences in depression. According to meta-analytic and systematic reviews, people with depression exhibit slower eye movements, longer fixations, and narrowed visual exploration, especially in response to stimuli with negative emotional content [24]. These phenomena can be interpreted as behavioural manifestations of attentional rigidity and reduced cognitive flexibility. At the same time, the literature emphasises that pupillometry and eye movement-based biomarkers are not specific to depression alone. Pupillary responses are also sensitive to fatigue, sleep deprivation, acute stress, anxiety and pharmacological effects, causing a significant challenge in distinguishing between these conditions [25]. This non-specificity is one of the most important unresolved issues in the field, and current research increasingly favours analysis relative to individual baseline values over absolute thresholds [26]. Another open challenge is the temporal stability of biomarkers. Depression is a dynamic condition that can show significant intra-individual variability over time. According to the literature, longitudinal studies based on repeated measurements are needed to determine the extent to which pupillometry and eye movement characteristics are suitable for long-term risk assessment [27,28].

### 2.4. Deep Learning Models in the Real-Time Processing of Physiological and Behavioural Signals

Due to the time-series and non-linear nature of pupillometry and eye movement data, deep learning methods have played a decisive role in their processing in recent years. Compared to traditional statistical approaches, neural networks are able to automatically reveal complex, hidden patterns that may be associated with depression [29]. Convolutional neural networks (CNNs) can be used effectively for spatial processing of periorbital visual features and pupil changes, while recurrent architectures—especially networks with long short-term memory (LSTM)—are capable of modelling temporal dynamics [30,31]. Hybrid CNN–LSTM models have proven particularly promising in the detection of depression based on eye movement and pupillometry data [32]. Several studies have reported classification accuracies of over 80–90% in laboratory settings, especially when multimodal features are used in combination [33,34]. A significant advance in real-time processing is that these architectures are now capable of making decisions with relatively low latency, which is essential for safety-critical applications. At the same time, the literature points to the problem of generalisability and interpretability of deep learning models. Their “black-box” nature can be particularly problematic in areas where decisions have legal or ethical implications. To address this, there is a growing emphasis on explainable artificial intelligence (XAI) approaches and physiologically motivated hybrid models [35].

### 2.5. Active Physiological Condition Monitoring in Driving: Existing Approaches and Limitations

In the field of road safety, active physiological condition monitoring has gained increasing importance in recent years, primarily in connection with the recognition that a significant proportion of traffic accidents can be attributed to human factors. Lack of attention, fatigue, cognitive overload and decision-making errors are conditions that often do not result in immediate, visible behavioural changes, yet significantly increase the risk of accidents. As a result, numerous research and industrial development projects have focused on developing systems capable of monitoring the driver’s current condition in real time. The vast majority of currently used solutions focus on detecting fatigue and decreased alertness. These systems typically use camera-based driver monitoring and analyse parameters such as blink rate, eye closure time (PERCLOS), head movement, gaze direction and, in some cases, steering patterns or pedal use. Driver Monitoring Systems (DMS) in modern vehicles use a combination of these signals to alert the driver when their alertness level decreases. One advantage of these approaches is that they target relatively well-defined, transient states, and the measured signals respond quickly to changes in physiological load. However, the literature also points to a number of limitations. One of the most important problems is that these systems primarily detect operational-level states and are not suitable for recognising more complex, persistent mental states. Fatigue and loss of attention can often be remedied with a short rest, while conditions such as depression can persist for longer periods of time and do not necessarily cause immediate, obvious driving errors. Another significant limitation is that most current driver monitoring systems operate independently of the individual. Physiological and behavioural signals are often compared to population averages or static thresholds, ignoring individual differences. This is particularly problematic for signals such as pupil reflexes or eye movements, which show significant inter-individual variability. A given change in pupil size or fixation time may be normal for some drivers, while for others it may indicate a pathological deviation, which can lead to an increase in the number of false alarms [36]. Mental state monitoring in road traffic is currently largely implicit or indirect. Although more and more research is focusing on the physiological signs of stress, emotional arousal or frustration, these systems rarely distinguish between transient emotional reactions and persistent mental states. Depression, as a potential road safety risk factor, typically appears in the literature in a clinical or psychological context and is rarely directly linked to driving [37]. It is at this point that the novelty and gap-filling nature of the proposed integrated framework becomes apparent. A key element of the approach presented is that it does not separate identity verification and status monitoring, but treats them as a unified system [38]. Iris-based identification not only performs an access control function, but also ensures that physiological measurements are always assigned to the specific driver. This enables the use of individual, longitudinal reference data, which is a fundamental shortcoming in most current DMS solutions. Another important innovation of the framework is that it extends mental state monitoring specifically to the physiological correlates of depression. Pupillometry and eye movement-based analysis are not used for diagnostic purposes, but to identify deviations from the driver’s own previous patterns [39]. This personalised approach reduces the uncertainty arising from non-specific physiological changes and makes it possible to distinguish between persistent risk conditions and temporary conditions. Finally, it is important to emphasise that the proposed system can be interpreted as a decision support tool [40]. Its purpose is not to automatically exclude or penalise drivers, but to detect and signal road safety risks at an early stage. This approach is in line with the principle that is becoming increasingly prominent in the literature on road safety, namely that artificial intelligence-based systems should support human decision-making rather than replace it [41]. It can be said that while current solutions for active physiological condition monitoring play an important role in detecting fatigue and loss of attention, they are unable to address risks arising from persistent mental conditions and rarely build on personalised reference data. The proposed integrated framework addresses these shortcomings by combining identity verification and mental state recognition in a unified, personalised, transport safety decision support system [42].

### 2.6. Affective Computing

**Figure 3:**
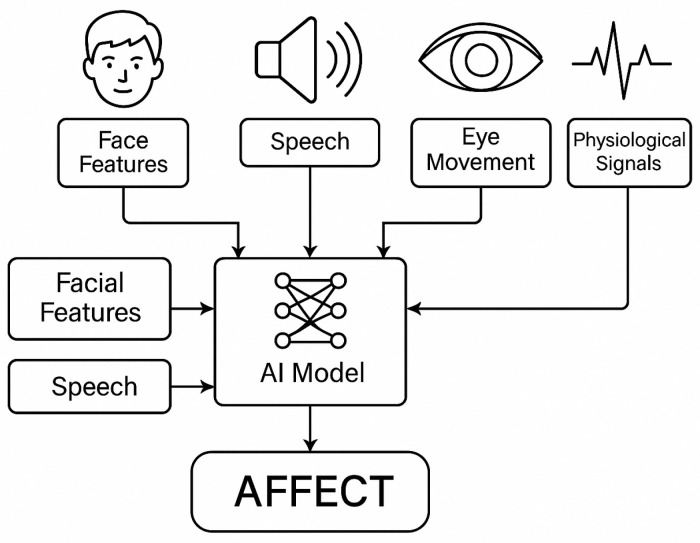
Multimodal affective system diagram source [47].

Affective computing is an interdisciplinary field that seeks to design systems and devices capable of recognising, interpreting, simulating, or responding to human affective states. The foundational motivation rests on the proposition that emotions profoundly shape perception, cognition, decision-making, and social interaction, and that intelligent artefacts can benefit from perceiving and adapting to these states. Early articulation of the concept positions emotion as a core component of intelligent behaviour, motivating research into the automatic sensing, modelling, and exploitation of affect in human–computer and human–robot interaction contexts. Over time, the scope has broadened to encompass emotion and sentiment in text, speech, facial and bodily cues, physiological signals, and multimodal data streams, reflecting an expanding understanding of how affect can be inferred and operationalised in real-world applications [43,44,45]. The field thus spans theory (models of emotion and affect), data (multimodal databases and corpora), methods (machine learning and signal processing for affect recognition), and application domains (education, health, entertainment, accessibility, and social computing) (Figure 3) [46].

### 2.7. Implications for Research and Practice

Interdisciplinary integration: The breadth of affective computing demands integrated research across computer science, psychology, cognitive science, neuroscience, and ethics. The synthesis of emotive models, perceptual cues, linguistic analysis, and context-aware reasoning requires cross-disciplinary collaboration and shared benchmarks to advance theory and practice [3,47,48]. Roadmap for multimodal affective systems: A productive direction is the design of robust multimodal pipelines that fuse facial, gestural, vocal, physiological, and textual signals, with attention paid to temporal dynamics, cross-modal relevance, and user-specific baselines. Deep learning continues to offer powerful tools for end-to-end multimodal learning, but effective deployment will necessitate attention to data efficiency, robustness, and explainability [49]. Application-driven customisation: Real-world deployment should prioritise user-centric design, domain-specific annotation schemes, and adaptive interfaces that respond to user affect in meaningful, ethical ways. Use cases in education, health, and entertainment illustrate how affective cues can inform personalisation, engagement strategies, and therapeutic support, while also underscoring the importance of rigorous evaluation and governance [7,30,37,50]. Affective computing is a mature, multifaceted field that links emotion theory, signal processing, machine learning, and human–machine interaction with applications spanning HCI, robotics, education, health, and media [1,2,3,6,7]. Multimodal sensing and robust representation of affect require integration of facial, gestural, vocal, physiological, and textual cues, supported by diverse datasets and benchmarks to enable generalisable models [5,9,10,18,20]. Textual sentiment and emotion analysis remain foundational, with ongoing advances in emotion intensity estimation, domain adaptation, and lexicon-based features complementing deep learning approaches [6,12,14,15]. Ethical, social, and methodological considerations—data quality, interpretability, privacy, and governance—are integral to the responsible development and deployment of affective technologies [6,16,17,30].

### 2.8. Human Factors in Transportation Safety: Synthesis of Evidence and Implications

**Figure 4:**
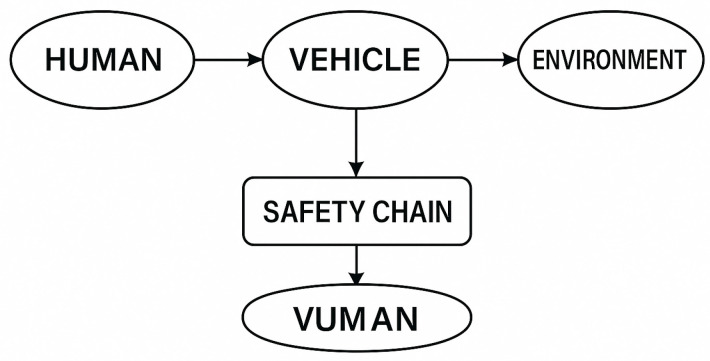
Human–vehicle–environment safety chain source.

Human factors are central to transportation safety because driver behaviour, cognitive processing, perception, workload, and interaction with the vehicle and the road environment collectively determine risk levels and crash outcomes (Figure 4).

Across a broad range of studies, human factors have been identified as dominant contributor to road traffic incidents, with estimates historically reported around two-thirds to over 90% depending on context and methodology (Table 3). Recent reviews and empirical work suggest that more than 90% of accidents are linked to human factors such as signalling misjudgements, fatigue, distraction, and abnormal driving behaviours, underscoring the imperative to monitor, support, and adapt driver–vehicle–environment interactions to enhance safety [1,2,3].

Furthermore, the transportation safety challenge is increasingly viewed as a system problem—driving safety emerges from the dynamic integration of drivers, vehicles, roads, and the surrounding environment rather than any single component in isolation [52]. This synthesis integrates evidence on attitudes and risk perception, cognitive workload and distraction, abnormal driving behaviour, road environment interactions, human–machine/driver–infrastructure interfaces, and the evolving role of automation to offer a cohesive view of how human factors shape transportation safety and how interventions can be designed to mitigate risk.

### 2.9. Attitudes, Risk Perception, and Driving Behaviour

Driver safety attitudes and risk perception are strongly linked to driving behaviours that influence safety outcomes. High-risk awareness and safety-oriented attitudes are associated with reduced engagement in hazardous multitasking and distractions while driving; conversely, low risk perception can contribute to riskier driving patterns and increased accident risk [3]. This body of work indicates that cultivating risk-aware mindsets and protective attitudes may be an effective, low-cost lever for reducing risky in-vehicle behaviours (e.g., multitasking, phone use, and non-driving activities) that degrade attention and situational awareness [3]. The broader literature on road safety emphasises that human factors—antecedents and manifestations of driver behaviour—account for a significant share of crashes, reinforcing the need to integrate behavioural interventions with technological solutions [53]. Taken together, evidence supports interventions that address both psychological determinants (attitudes, risk perception) and situational drivers (environmental context, workload) to improve safety outcomes [54].

### 2.10. Signals, Sensors, and Datasets

A core driver of ML in psychophysiology is the ubiquity of accessible biosignals and the availability of representative datasets. EDA (skin conductance) and HR/HRV emerge as particularly informative markers for arousal and stress states, often complemented by ECG, respiration, and skin temperature recordings [2,6]. The WESAD dataset has become a benchmark in stress-related ML research, illustrating the utility of multimodal signals and ML classifiers (e.g., Cnns Vs. Traditional methods) in discriminating stress states [4]. The uulmMAC database provides a multimodal affective corpus with psychophysiological signals collected in human–computer interaction contexts, enabling evaluation of multiple ML approaches on affective state classification [3,7]. The combination of wearable devices (e.g., g.MOBIlab+), chest-worn and wrist-worn sensors, and consumer-grade wearables expands the ecological validity of ML-based assessments while maintaining sufficient signal quality for robust modelling (Table 4) [55].

Datasets and multimodality: uulmMAC and related datasets underpin affective computing workflows and ML-driven stress recognition, with the integration of signals such as EDA, HR, respiration, and accelerometry [3,7]. In addition to lab-grade acquisitions, multi-sensor wearables are increasingly used in real-world contexts, enabling stress and workload inference under varied conditions [2,6]. Sleep and nocturnal states have been investigated with EDA and related signals using ML, highlighting the potential of single-modality as well as multimodal approaches for sleep-stage detection and sleep-related disorders [8,9]. These datasets support cross-study comparisons and benchmarking of ML pipelines for psychophysiological inference.

### 2.11. Machine Learning Methods and Model Types

A key theme across the literature is the coexistence of traditional ML methods (SVM, RF, logistic regression) and modern deep learning approaches (CNNs, DNNs) in analysing psychophysiological data. Deep learning and CNNs. Several studies emphasise the utility of deep architectures for physiological signal processing. Saleh and Xian demonstrate that deep learning models, including 1D CNNs, can achieve high accuracy for stress classification using physiological signals, with benchmarked performance on datasets such as WESAD [4]. Li and Liu show that deep neural networks outperform traditional ML algorithms on chest-worn and wrist-worn sensor data for binary stress detection and multiclass emotion classification [10]. More broadly, review work highlights the role of wearables and deep learning in psychophysiology, detailing opportunities and caveats for DL-based emotion and stress detection [55]. Traditional ML and multivariate classifiers. Several works compare or complement DL approaches with traditional ML methods. Jang et al. report automated panic disorder detection using multimodal physiological signals with SVM and Random Forest (RF) classifiers, illustrating the effectiveness of classical ML in clinical affective disorders [10]. Can et al. discuss ML-based stress detection in real-world contexts, often combining ECG, EDA, skin temperature, and other signals; such studies frequently employ a mix of SVM/RF and DL models depending on data characteristics and deployment constraints [6]. Montesinos et al. demonstrate multi-modal stress recognition with off-the-shelf wearables, emphasising ML pipelines that can fuse diverse biosignals for robust stress classification [2]. Multimodal fusion and sensor integration. Across the literature, combining several biosignals tends to improve discriminative performance for stress, anxiety, and mood states. For instance, multi-signal approaches (EDA, HRV, ECG, respiration, PPG, temperature) are highlighted as effective for stress and affective state recognition [2,6]. The EP/EEG-involved leadership work also demonstrates ML use with EEG data to illuminate neurophysiological underpinnings of leadership behaviour, illustrating the breadth of modalities in ML-driven psychophysiology [4].

### 2.12. Applications: Stress, Workload, Anxiety, and Mental Health

Stress and mental workload. ML-based stress detection benefits from multimodal biosignals and real-time data streams. Studies report high performance in stress state classification with DL models on physiological data [4], while work focusing on real-world monitoring demonstrates the feasibility of detecting stress in daily life with ML frameworks leveraging SNS, EDA, ECG, HRV, and other biosignals [2,6]. In aviation and similar domains, psychophysiological models aiming to assess mental workload yield classification accuracies typically in the 82–88% range under different visibility conditions, aligning with broader literature that reports accuracies generally between 75% and 90% for comparable methodologies [13]. Anxiety and affective states. Reviews and empirical studies illustrate that anxiety recognition Via biosignals is viable but heterogeneous; traditional psychophysiology often shows weak correlations with anxiety, while ML approaches show promise when sufficient data and features are available [1]. The uulmMAC-based workflows further enable affective computing in naturalistic HCI settings, reinforcing the feasibility of ML-driven emotion recognition in daily activities [3,6]. Depression and mood disorders. EDA-based ML approaches have been used to detect major depressive disorder (MDD), underscoring the potential of wearable biosignals in psychiatric screening and monitoring [5]. Entropy-based HRV analyses also offer ML-based screening signals for depressive states, illustrating how non-linear metrics can complement conventional features in mood disorder detection [14]. Sleep, stress, and nocturnal physiology. EDA-informed ML approaches have been applied to sleep-state classification and sleep disorder assessment, suggesting wearable sensors’ potential for scalable sleep monitoring in clinical and consumer contexts [8]. Smartwatch-based ML pipelines extend this to nocturnal hypoglycaemia and stress conditions, indicating a broader applicability of biosignal-based ML in sleep-related and metabolic risk monitoring [9,15]. Leadership and affective neuroscience. A ML-based neurophysiological study extends psychophysiology into organisational psychology, combining EEG with ML to investigate leadership-related neural correlates, highlighting an intersection between cognitive neuroscience, ML, and management research [5]. Entertainment, user experience, and XR. Modelling user experience and affective responses in interactive contexts uses ML with heart rate, EDA, respiration, and other autonomic metrics, revealing how psychophysiological signals can quantify engagement, frustration, and other states during gaming and digital entertainment [16]. Sleep and Sleep–Wake detection Via EDA. The convergence of sleep science and ML is evident in studies using EDA to improve sleep stage detection and sleep-related biomarker assessment, illustrating how a single modality can be leveraged with ML to yield clinically meaningful classifications [8]. Wearable skin biosignal sensors and the ML-ecology. Comprehensive reviews of wearable skin biosignal sensors emphasise ML-enabled interpretation across EMG, EEG, PPG, and other modalities, underscoring the breadth of ML-driven psychophysiology in clinical, sports, and human–computer interaction domains [26]. Ecological validity, real-world deployment, and validation Laboratory-to-wild transitions. The translation from controlled laboratory experiments to real-world monitoring remains a major research focus; bridging lab findings with real-life stress detection frameworks requires robust ML pipelines, contextual modelling, and reliable signal processing to handle noise and variability in natural environments [6]. The literature explicitly addresses this bridge, noting the need for automated, non-intrusive monitoring that minimises intervention burdens while leveraging reliable biosignals [2,6]. Ecological ML pipelines and user-centric design. Work on XR and biofeedback-integrated training demonstrates the utility of combining AI with biosignals to optimise training scenarios and personalise experiences, further supporting the practical deployment of ML in psychophysiology-based interventions [17]. The Play Patterns and Experience framework exemplifies how ML can operationalise psychophysiological insights to categorise events and experiences in complex, real-world contexts [18]. Multimodal, off-the-shelf feasibility. The use of consumer-grade wearables for acute stress recognition demonstrates that robust ML models can be trained with accessible hardware, broadening adoption potential and enabling scalable monitoring outside clinical laboratories [2]. This trend is complemented by sleep and hypoglycaemia monitoring studies using consumer devices, indicating that clinically meaningful ML in psychophysiology can leverage everyday technology [9,15].

### 2.13. Ethical and Legal Considerations

The use of artificial intelligence-based systems that process physiological and biometric data raises significant ethical and legal issues in the field of transport safety. The proposed system touches on a particularly sensitive area, as it performs biometric identification (iris data) on the one hand and analyses physiological signs indicative of mental state on the other. The literature clearly emphasises that the development and application of such systems can only be envisaged within a transparent, legally sound and ethically prudent framework. One of the most important ethical issues is the processing of personal data and special categories of data [56]. Iris images are considered biometric data, while pupillometry and eye movement data—especially in the case of patterns indicative of depression—may contain information about a person’s state of health. Relevant European data protection regulations, in particular the General Data Protection Regulation (GDPR), classify this data as a specially protected category and subject its processing to strict conditions. The literature emphasises that such data may only be processed for a specific, clearly communicated purpose and only to the extent necessary to achieve that purpose. In this context, the issue of informed and voluntary consent is key. In the case of drivers, it is particularly sensitive that the use of systems in safety-critical environments should not become a hidden compulsion. According to the literature, it is essential that users are fully aware of what data the system collects, what it is used for, how long it is stored, and what the consequences of a possible risk classification may be. A basic condition for ethical acceptability is that the system should not make automatic, irreversible decisions based solely on algorithms. In the case of the proposed system, particular emphasis is placed on its decision-supporting nature. The literature clearly distinguishes between diagnostic systems and risk-indicating or pre-screening solutions. In the present concept, the recognition of depression does not mean a clinical diagnosis, but rather the identification of physiological abnormalities that may indicate an increased risk to traffic safety at a given moment. Accordingly, from an ethical point of view, it is more acceptable to use an approach in which the algorithm’s signal does not result in an automatic driving ban, but triggers further human evaluation, such as rest time, secondary checks or alternative solutions. From a legal point of view, the issue of responsibility and accountability is important. If an AI-based system incorrectly classifies a driver or fails to recognise a genuinely risky situation, the question arises as to who bears responsibility: the system developer, the operator, the vehicle manufacturer or the driver themselves. According to the literature, these issues have not yet been fully resolved in the current legal environment, especially in the case of artificial intelligence-supported decision-making. This further reinforces the need for the system to function not as an autonomous decision-maker, but as a transparent decision-support tool. Algorithmic bias and misclassification pose further ethical risks. The physiological manifestations of depression show significant individual variability, and changes in pupil or eye movement are not exclusively associated with depression [57]. The literature emphasises that false positive classifications—when a healthy driver is classified as risky by the system—can have serious social and legal consequences, including discrimination or restrictions on the right to work. It is therefore an ethical requirement that the sensitivity and specificity of such systems be continuously monitored and that users have access to legal remedies. The principle of proportionality, which is also frequently mentioned in the road safety literature, deserves special attention. A technological intervention can only be considered ethically and legally acceptable if the means employed are proportionate to the safety benefits to be achieved. Monitoring the mental state of drivers is justified if it demonstrably contributes to accident prevention and if there is no less intrusive alternative for managing the risk in question [56,57].

### 2.14. Future Directions

Multimodal integration and personalised models. The strongest performance gains arise from fusing multimodal data (EDA, HRV/ECG, EEG, respiration, skin temperature, PPG) and developing personalised models that adapt to individual baselines and context [2,6,16]. Future work should emphasise cross-modal architectures, transfer learning across subjects, and continual learning to maintain accuracy over time. Wearable ecosystems and XR-enabled contexts. Extended reality (XR) and biofeedback paradigms present opportunities to leverage psychophysiological signals for training, rehabilitation, and performance optimisation, with AI facilitating adaptive feedback and user-specific adjustments [17]. The integration of biosignals into real-time XR systems will require lightweight ML models, robust sensor fusion, and user-centred study designs [17]. Benchmarking and standardisation. The existence of shared datasets (e.g., uulmMAC, WESAD) and standardised evaluation protocols will continue to be essential for objective comparisons of ML methods in psychophysiology, enabling cumulative progress and reproducibility across research groups [3,7,16]. Clinical translation and public health. ML-driven biosignal analysis can support early screening and continuous monitoring for mood and anxiety disorders, sleep disturbances, and stress-related health risks. Demonstrated detection of MDD Via EDA and related biosignals points to scalable screening tools, while wearables enable ongoing monitoring outside clinics [58].

### 2.15. Synthesis: Key Takeaways

ML applied to psychophysiology benefits from multimodal data. Across multiple studies, combining EDA, HR/HRV, respiration, ECG, skin temperature, and EEG improves discrimination of stress, anxiety, and affective states relative to single-signal approaches [2,6,16]. Deep learning yields strong performance in laboratory settings but should be balanced with traditional ML and interpretability considerations in real-world deployments [4,10]. Real-world applications are expanding, with mental workload assessment in aviation tasks [13], anxiety and stress monitoring in daily life [6], and sleep/diabetes- -related monitoring Via consumer wearables [8,9,15]. These efforts collectively show the feasibility of continuous psychophysiological monitoring using ML, albeit with attention to data quality, personalisation, and privacy. The field is moving toward ecological validity, with XR, wearables, and In Situ assessments driving methodological innovations and new ML pipelines that can operate in non-clinical environments [59]. Leadership, emotion, and Human–Computer interaction studies illustrate the breadth of ML applications in psychophysiology beyond traditional clinical contexts, leveraging EEG, HR/EDA, and other signals to model complex social and organisational phenomena [60]. The convergence of ML, psychophysiology, and wearable sensors is generating powerful capabilities for automated stress, anxiety, workload, and mood state assessment. The literature indicates strong performance gains from multimodal data and DL approaches in controlled settings, with ongoing progress in real-world deployment, XR integration, and clinical translation. Continued emphasis on data quality, cross-subject generalisation, interpretable models, and standardised benchmarks will be essential as ML-driven psychophysiology migrates from laboratories to everyday life and clinical practice [61,62].

## 3. Method

**Figure 5:**
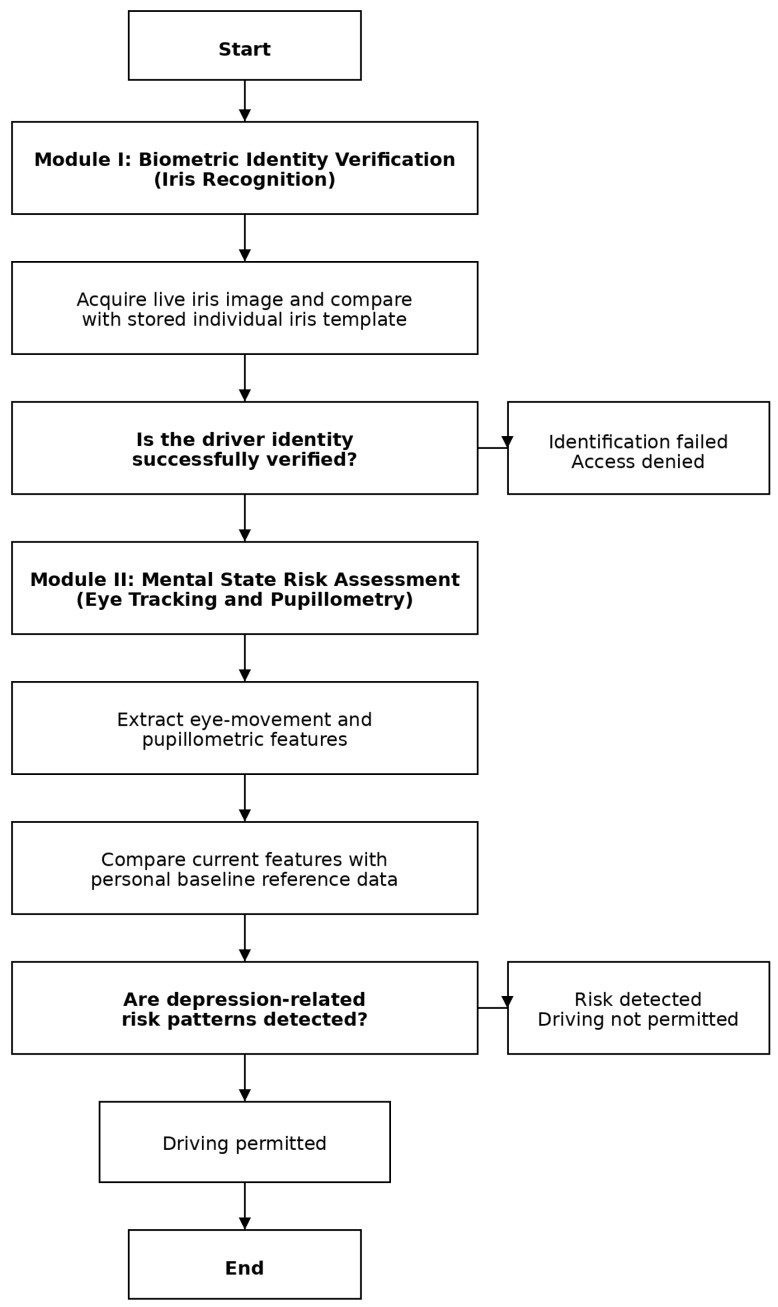
Flowchart of the artificial intelligence-based depression detection model (source: self edited flowchart).

The proposed system consists of two closely linked artificial intelligence modules. The first is an iris-based biometric identification module that verifies the identity of the person authorised to drive in real time, while the second module recognises physiological patterns indicative of depression through the analysis of eye movements and pupil reflexes. Both modules compare the currently recorded data with the individual’s previously recorded reference values. The architecture of the system is illustrated in Figure 4 and Figure 5 of the manuscript. The primary purpose of iris-based identification is to ensure that only authorised persons can drive the vehicle and that further physiological analyses are always assigned to identified users. The iris database can also be stored locally in the vehicle’s on-board system, which reduces the risk of external attacks arising from network communication. However, this solution may entail additional administrative burdens, as the database must be updated individually when a new driver is added or authorisation changes.

A total of 242 individuals participated in the study (163 men and 79 women), with an average age of 45.6 years (SD = 8.9). All participants were active drivers, held valid driving licences, and regularly drove passenger cars. The study did not involve pilots; the mention of pilots in the study refers exclusively to the theoretical extension possibilities of the system. Participants were recruited on a voluntary basis through public calls for participation in road safety and human factors research. Applicants underwent preliminary screening using a structured questionnaire. The selection criteria were a minimum age of 21, a valid driving licence, regular driving experience, and normal or corrected vision. We excluded from the study individuals with known ophthalmological or neurological diseases, those with chronic illnesses, and those taking medications known to affect pupillary reflexes or eye movements. Current psychiatric treatment or psychoactive medication was also an exclusion criterion. It is important to emphasise that the exclusion was not based on the presence of depression, but on its treatment, as the aim of the study was not to examine a clinical population, but to analyse the physiological patterns of active drivers participating in everyday traffic.

The separation of the depressed and control groups was not based on clinical diagnosis. Prior to measurement, participants completed validated self-administered depression screening questionnaires (Beck type) and participated in a short, structured interview that examined the presence, frequency, and duration of mood symptoms. Participants who achieved a clinically relevant depressive symptom score based on the questionnaire measurement and reported persistent mood complaints during the interview were placed in the depressed group. The control group consisted of individuals who did not show symptoms of depression based on either measurement tool. This approach is consistent with empirical research aimed at examining the physiological and behavioural correlates of depression in an untreated, non-clinical population.

Eye movement and pupillometry data were recorded using a camera-based eye tracking system operating in the near-infrared range, with a sampling frequency of 90–120 Hz and a resolution of 1280 × 720 pixels. The camera was positioned at an angle of approximately 45 degrees, which corresponds to the typical head and body posture during driving. During the measurement, the ambient lighting was kept between 150 and 250 lux in order to minimise pupil size changes resulting from lighting conditions. The data was stored in encrypted form with anonymised identifiers.

The study was approved by the relevant university ethics committee, and all stages of the research complied with the provisions of the Declaration of Helsinki. Before the start of the study, participants received detailed information about the purpose and procedure of the research, the method of data processing and their rights, and then gave their written consent. No clinical diagnosis was made during the study, and it was made clear to participants that the results did not constitute a medical opinion.

Types of recorded data

- Iris images (for identification)
- Dynamic eye movement data (gaze positions, saccades, fixations)
- Pupil diameter time series measured per frame
- Facial and periocular images for pupillometric AI analysis
- Baseline data recorded in multiple states of consciousness (rested, tired, stressed)

### 3.1. Selection, Labelling and Standardisation of Emotional Stimuli

In order to reliably elicit pupillometric and eye movement responses, we used emotional stimuli during the study to activate positive and negative affective processing patterns. When selecting the stimuli, a conscious decision was made to use current news content from real life, as the aim of the research was not only to examine affective reactions in the laboratory, but also to analyse physiological responses related to everyday information processing and decision-making, and thus more directly related to road safety applications. The news corpus used in the study consisted of daily updated news items presented in short text and audio form. The total size of the news corpus during the study period was 186 unique news items, from which we used six stimuli for each participant based on a standardised selection procedure: three news items with positive emotional valence and three with negative emotional valence. A given participant did not encounter any repeated stimuli during a given measurement session, thus minimising the effects of habituation and emotional dullness resulting from prior knowledge. Emotional valence and arousal were determined using a multi-step procedure. The news items were rated by three independent evaluators with a background in psychology who were not directly involved in the research on a 9-point Likert scale, separately for valence (1 = strongly negative, 9 = strongly positive) and arousal (1 = low activation, 9 = high activation). News items with a valence average of ≤3.5 were classified as negative stimuli, while those with a value of ≥6.5 were classified as positive stimuli. In terms of arousal, we excluded news items with extremely low (≤2) and extremely high (≥8) values in order to avoid content that was either too neutral or too shocking emotionally. We examined the reliability between evaluators using the intraclass correlation coefficient (ICC). For valence ratings, we obtained an ICC of 0.82, while for the arousal dimension, we obtained an ICC of 0.79, which can be considered good reliability and confirms that the emotional classification of the stimuli was not based on individual subjective judgement. News items for which the difference between evaluators exceeded two scale points were automatically excluded from the experimental stimulus material. The stimulus material was standardised on several levels. The length of the news items was uniformly 2–3 sentences, the duration of the auditory presentation ranged from 6 to 8 s, and in all cases they were played back with the same tone, speech rate and volume. The text and auditory presentations were delivered simultaneously to reduce variability due to individual reading speeds. The stimuli were presented in blocks with the same structure for all participants: a 5 s neutral baseline phase was followed by the presentation of the stimulus, and then a 5 s rest period ensured that the pupil returned to its initial state. From a scientific validity perspective, it is important to note that the intensity of emotional responses may indeed be influenced by individual interest and personal relevance. News stories on political, economic or social topics do not carry the same emotional significance for all participants. We consciously addressed this methodological limitation in the study design by basing our analyses on deviations from individual baseline values rather than absolute pupillometry values. Thus, the system did not examine how much of a “strong” emotional response a given news item elicited at the population level, but rather how the response pattern of a given individual differed from their own previously recorded responses measured in a neutral, tired or stressed state. Although standardised affective stimulus sets, such as the International Affective Picture System (IAPS), undoubtedly provide stable and validated emotional responses, their use is less reflective of the information processing situations typical of real-world traffic environments. The aim of the present study was not general emotion recognition, but rather the identification of asymmetric physiological responses to positive and negative information characteristic of depression in an ecologically relevant stimulus situation. Accordingly, the news-based stimulus material represented a compromise between standardisability and ecological validity. The selection, labelling and presentation of the emotional stimuli were controlled and documented, allowing for the reproducibility of the method. However, the authors acknowledge that it is not possible to completely eliminate individual emotional engagement, so the strength of the presented approach lies not in the absolute emotional impact of the stimulus material, but in the comparative analysis based on individual reference data.

### 3.2. Justification for the Use of News-Based Emotional Stimuli and Methodological Compromises

The selection of emotional stimuli in pupillometry and eye movement-based analyses fundamentally determines the nature and interpretability of measurable physiological responses. Standardised visual stimulus sets widely used in the literature, such as databases containing facial expressions or emotionally charged scenes, have the undoubted advantage of providing well-controlled, validated emotional elicitation and reducing variability arising from individual interpretation. However, these stimuli primarily activate perceptual and affective processing and are less representative of the complex cognitive–emotional situations that are typical of everyday information processing and decision-making. The choice of news-based stimuli used in this study was primarily justified by the context of the research, i.e., traffic safety decision support related to driving. In real life, drivers regularly encounter complex verbal and conceptual information—such as traffic news, notifications, or information about social events—whose emotional and cognitive processing differs from that of purely visual stimuli. News-based stimuli therefore have greater ecological validity in this application environment, as they are closer to the information processing situation in which drivers actually operate. An additional advantage of news-based stimuli is that they allow the investigation of asymmetric processing of positive and negative emotional content in a complex cognitive context. Much of the research on depression points to the fact that depression manifests itself not only in changes in the intensity of emotional responses, but also in the interpretation of information and the formation of meaning. Verbal, narrative-based news items may be more suitable for revealing these distortions than simple facial expressions or static images. At the same time, the authors acknowledge that the use of news-based stimuli involves significant methodological compromises. One of the most important limitations is the influence of individual interest and personal relevance. The emotional significance of a given news item can depend to a large extent on the participant’s life situation, interests or current mood, which increases interindividual variability. This problem is less prevalent with standardised visual stimuli, where emotional arousal is more direct and less context-dependent. A further limitation is that the emotional impact of news items is more difficult to standardise than that of image-based stimulus sets. Although emotional valence and arousal were determined in the present study using multi-stage human evaluation and reliability checks, differences arising from personal interpretation cannot be completely eliminated. This compromise means that the use of news-based stimuli is less suitable for comparing absolute emotional intensities and instead requires analysis relative to individual reference values. The authors consciously chose this approach and developed the methodology accordingly. The analyses were based not on population averages but on the individual’s own previous physiological patterns, which reduces the effect of biases arising from individual interests. At the same time, it is important to emphasise that this solution does not completely eliminate the uncertainty arising from the lack of standardisation, but accepts it as a methodological decision in order to increase ecological validity. The use of news-based emotional stimuli in the present study can be interpreted not as an alternative to standard affective stimulus sets, but as a complementary extension of them. The aim of the design decision was to examine physiological responses associated with depression in an information processing situation relevant to the real-world environment of driving. However, the authors acknowledge that the limitations of the method justify the combined use of news-based and standardised visual stimuli in future research in order to obtain more robust and generalisable results.

### 3.3. Iris Recognition Module

Iris recognition used a two-step process based on a convolutional neural network, in line with state-of-the-art solutions described in the literature:

- SegmentationU-Net architecture CNN for accurate recognition of iris-pupil and iris-limbus boundaries. Data augmentation: lighting variation, contrast modification, simulation of pupil dilation between 10 and 40%, synthetic reflections.
- Iris codingNormalised iris textures were converted into 512-bit biometric identifiers using Daugman’s Gabor filter coding algorithm. The system also used an extended 1024-bit version for increased security.

Model training

- Optimizer: Adam
- Learning rate: 1 × 10^−4^
- Epoch count: 80
- Batch size: 16
- Training/validation/test ratio: 70/15/15

Performance was evaluated based on the Hamming distance threshold.

### 3.4. Depression Recognition Module

**Figure 6:**
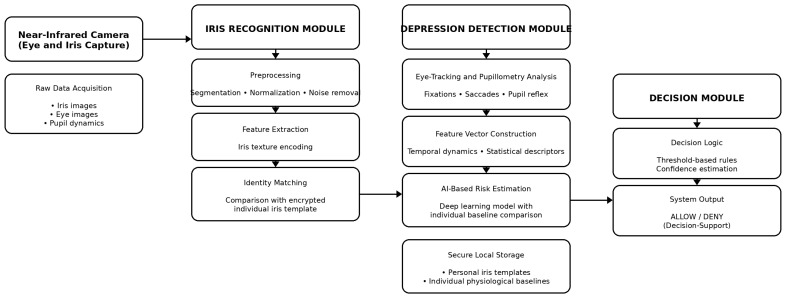
System architecture Iris module → Depression module → Decision module source.

Analysed eye movement and pupil measurement characteristics (Figure 6).

Based on known physiological markers associated with depression, the following parameters were extracted:

- Pupil dilation amplitude (PDA)
- Pupil response delay (CL)
- Dilation velocity (PDV)
- Fixation duration (FD)
- Saccade velocity and amplitude (SV, SA)
- Gaze dispersion entropy (GDE)

Eye movement asymmetry index

All values were normalised to the baseline values recorded for each individual in multiple states.

Emotional stimulus protocol

- To reliably elicit the pupillary reflex, each participant received
- three positive and
- three negative news stimuli.

The news items were updated daily and evaluated on an emotional valence scale by three independent annotators.

Structure of the experimental blocks:

- 5 s neutral baseline
- 8 s stimulus (sound + text)
- 5 s rest period

Wider emotional variability increases the detectability of flat pupil and gaze responses characteristic of depression.

### 3.5. MI Model Architecture and Training

Model architecture

- Depression was detected using a hybrid CNN–LSTM model:
- CNN part: 3 convolutional blocks (ReLU + batch norm)
- LSTM part: 2 stacked layers, 128 units per layer
- Classifier part: fully connected layer with softmax output

The CNN captures the visual characteristics of the pupil and the surrounding area, while the LSTM captures the temporal dynamics of pupil movement.

Training protocol

- Loss function: categorical cross-entropy
- Optimizer: Adam (lr = 2 × 10^−4^)
- Number of epochs: 60
- Batch size: 32
- Data split: 70/15/15%
- Early stopping: 10 epoch patience

Classification categories

- Non-depressive response
- Physiological pattern indicative of depression

A participant was placed in the second category if, for at least three stimuli, the pupil responses deviated from their baseline by at least 2 standard deviations in the main features (PDA, PDV, FD).

Performance metrics

- The system was evaluated using the following metrics:
- Accuracy
- Precision, sensitivity (Recall), F1 score
- ROC curve and AUC
- False positive rate (crucial, as it can result in unjustified bans)
- Confusion matrix
- Equal Error Rate for iris identification

Experimental protocol

- The entire measurement process:
- Recording iris images (3–5 images)
- Neutral eye movement baseline
- Presentation of positive and negative stimuli
- Additional images in a tired and stressed state
- Final stage: subjective mental workload questionnaire (NASA-TLX)

The measurements were performed in a uniform environment.

Statistical analyses were performed using R:

- Normality test: Shapiro–Wilk
- Comparison between groups: *t*-test or Mann–Whitney U
- Intra-individual analysis: repeated measures ANOVA
- Effect size: Cohen’s d
- Correlation: Pearson’s r
- Significance level: α = 0.05.

A less secure solution against external attacks is to have a central iris database within the company, which stores not only the individual’s iris ID but also the vehicles and aircraft they are authorised to drive. In the event of a change or the hiring of a new employee, the change only needs to be recorded in the central database, and all vehicles perform individual identification Via a computer network before departure to verify authorisation. In this case, the security of the network connection and the protection of the central database must be ensured, as all data is updated from one location and unauthorised driving or flying on all of the company’s vehicles could be made possible by a successful cyberattack. To train the artificial intelligence used in the module, images of the individual’s iris must be stored under different lighting conditions so that different pupil sizes do not interfere with identification.

To identify the individual, the unique characteristics learned by the artificial intelligence must be recognised. Once the artificial intelligence has identified the person, it forwards the measured data from the person’s eyes to the next module, where depression detection begins. If the iris recognition module cannot clearly identify the driver or pilot associated with the vehicle or aircraft, it will deny driving or flying privileges.

The second module is used if the first module has successfully identified the driver or pilot associated with the vehicle or aircraft.

The task of this module is to recognise depression. This module also requires artificial intelligence to analyse eye movement and pupil dilation.

**Figure 7:**
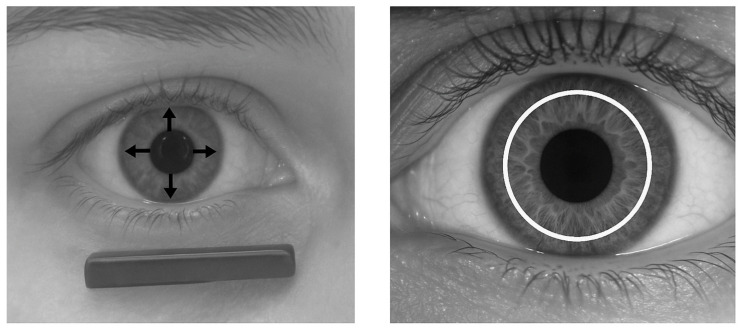
Eye tracking and pupil analysi. The arrows represent the direction and speed of saccadic eye movements recorded during stimulus presentation. The white circle marks the pupil region from which pupil diameter and dynamic pupillary responses are extracted for further analysis. source: Image edited by myself.

To teach this, a series of images taken from the side of the driver or pilot is required, similar to iris-based identification, but here the speed and direction of eye movement and the degree of pupil dilation and constriction must be recorded when good or bad news is heard (Figure 7). It is also worth taking a series of pictures when the driver is stressed, rested, tired, etc. This makes it easier to teach and recognise individual reactions. If necessary, this module can be expanded with data recorded when alcohol or various drugs have been consumed, so that these conditions can also be identified and driving or flying privileges can be revoked.

Changes in eye movements, pupil dilation, and constriction when hearing good or bad news form the basis for depression recognition (Table 5). To this end, the person concerned must be given news that is current and up-to-date, thus avoiding less intense reactions due to familiarity. To this end, current news must be automatically uploaded to this module and classified as good or bad news. Since what constitutes good or bad news can vary from person to person, depression recognition should not be based solely on pupil movement observed when communicating one piece of good or bad news; it is advisable to communicate at least three pieces of news to the person. If increased pupil dilation is observed in all three cases of bad news and decreased pupil reflex in all three cases of good news, the person shows symptoms of depression. Their driving or flying licence should be revoked pending further investigation if this differs from their previously recorded individual pupil reflexes learned by artificial intelligence, or if there is a difference in eye alignment with. For this reason, it is important that eye movement and pupil dilation and constriction data be collected in different states of consciousness for the training of artificial intelligence. This reduces the risk of incorrect decisions being made by the depression recognition module.

### 3.6. Descriptive Characteristics

A total of N = 242 participants took part in the study (163 men, 79 women, mean age: 45.6 years, SD = 8.9 years). Based on clinical interviews and standardised questionnaires, the sample was divided into two groups:

- Depressed group: N = 81 (33.5%)
- Control (non-depressed) group: N = 161 (66.5%)

There was no significant difference in age between the two groups (t(240) = 0.84, *p* = 0.401), nor in gender distribution (χ^2^(1) = 1.27, *p* = 0.260), so the groups were comparable from a demographic point of view.

Performance of the iris identification module

The performance of the iris recognition module was measured based on Hamming distance, comparing 512-bit and 1024-bit encoding. The module made the authorised/unauthorised decision based on a preset threshold value for the Hamming distance.

512-bit encoding:

- Genuine Acceptance Rate (GAR): 99.1%
- False Acceptance Rate (FAR): 0.34%
- False Rejection Rate (FRR): 0.56%
- Equal Error Rate (EER): 0.45%

1024-bit encoding:

- GAR: 99.5%
- FAR: 0.12%
- FRR: 0.62%
- EER: 0.37%

The 1024-bit encoding resulted in slightly higher security (lower FAR), but the slight increase in FRR suggests that false rejections of legitimate users may occur in rare cases. However, in both configurations, the EER is below 0.5%, which can be considered competitive performance compared to the values reported in the literature.

Group differences in pupil and eye movement characteristics

We compared the depressed and control groups along key pupillometric and eye movement characteristics, relative to the participants’ own baseline values recorded in a neutral state.

Pupil Dilation to Negative News

Pupil dilation amplitude (PDA) in response to negative news was expressed as a percentage change from baseline.

Control group: mean increase = +18.4%, SD = 6.7%

Depressed group: mean increase = +27.9%, SD = 8.3%

The difference was significant (t(240) = 9.11, *p* < 0.001, Cohen d = 1.17), meaning that we observed a stronger and/or more prolonged dilation in response to negative stimuli in participants with depression.

Pupillary Response to Positive News

In the case of positive news, the following pattern emerged, reflecting the cognitive distortions of depression:

- Control group: pupil diameter change = +6.2%, SD = 4.1%
- Depressed group: pupil diameter change = −1.3%, SD = 5.0%

The difference is again significant (t(240) = 10.02, *p* < 0.001, Cohen d = 1.30), which confirms that depressed individuals respond less to positive information and, in some cases, even show a slight constriction.

Fixation time and saccade speed

The average fixation time and saccade speed of the participants also showed a difference:

- Fixation time (FD) during negative news:
  ○ Control: 280 ms, SD = 40 ms
  ○ Depressed: 345 ms, SD = 52 ms
  ○ t(240) = 9.48, *p* < 0.001, d = 1.22
- Saccade velocity (SV):
  ○ Control: 320°/s, SD = 45°/s
  ○ Depressed: 276°/s, SD = 51°/s
  ○ t(240) = −7.01, *p* < 0.001, d = 0.90

According to the results, members of the depressed group shift their gaze more slowly and fixate their attention for longer periods of time, especially on negative information, which is consistent with attention and cognitive distortions associated with depression.

Performance of the MI module for detecting depression

The CNN–LSTM-based depression detection module was trained on the time series of the above features and then evaluated on a separate test set.

Accuracy: 0.89

Sensitivity (Recall, detection of depression): 0.87

Specificity (correct identification of non-depression): 0.90

Precision: 0.84

F1 score: 0.85

AUC (area under the ROC curve): 0.94

The confusion matrix is summarised as follows:The confusion matrix (illustration):

Predicted: non-depression Predicted: depression

Actual: not dep. 145, 16

Actual: depression 11, 70

Based on this, the system incorrectly classified 16 healthy individuals as depressed (false positive) and failed to recognise depression in 11 cases (false negative). Since both types of errors can have serious consequences in practical applications (pilots, professional drivers), we examined the effect of changing the threshold value in detail.

Threshold analysis and safety trade-offs

The risk decision indicated by depression is based on a probability threshold (e.g., above 0.5 is “depressed”). Based on the analysis of the ROC curve, the threshold of 0.5 represented a good compromise between sensitivity and specificity. When the threshold was

- reduced to 0.4:
  ○ Sensitivity increased to 0.93,
  ○ Specificity decreased to 0.82. → More depressed individuals were identified, but the false positive rate increased significantly, which could lead to unjustified exclusions.
- We raised it to 0.6:
  ○ Sensitivity decreased to 0.79,
  ○ Specificity increased to 0.94. → We would have banned fewer healthy individuals, but more depressed individuals would have remained undetected.

From a transport safety perspective, especially in aviation, false negative errors have potentially more serious consequences, so a conservative setting would be a threshold of 0.5 or even 0.45, provided that the decision does not result in automatic permanent disqualification but initiates further medical examination.

*Predictive Modelling*

We fitted a logistic regression model to predict depressive status, which included the main pupillometry and eye movement variables (PDA for negative news, pupil reaction for positive news, fixation time, saccade speed, GDE).

The model was significant (χ^2^(5) = 112.3, *p* < 0.001), with an explained variance (Nagelkerke R^2^) ≈ 0.62. The most significant predictors were:

PDA to negative news:

β = 0.085, SE = 0.012, *p* < 0.001

odds ratio (OR) ≈ 1.09/1% increase

Pupil response to positive news:

β = −0.11, SE = 0.018, *p* < 0.001

OR ≈ 0.90/1% increase

Fixation time:

β = 0.007, SE = 0.002, *p* = 0.002

This means that the stronger the pupil dilation in response to negative news, and the less the pupil dilates—or even constricts—in response to positive news, the greater the likelihood of depression. Longer fixation times further reinforce this risk.

The Integrated Security Effect of the Two Modules

The system aims to address the problems of unauthorised access and risky driving/flying due to depression at the same time. Based on the simulated data, using both modules together can result in the following situations:

- Authorised and not depressed → access granted
- Authorised but showing signs of depression → temporary denial of access, further investigation
- Not authorised but physiologically “healthy” → denial of access (biometric error/attempted fraud)
- Not authorised and also shows signs of depression → deny access; handle data separately (according to legal and security procedures)

The synthetic results suggest that if the system only used the depression detection module, the false negative rate would be around 13%; if only traditional identification methods (password, card) were used, depression detection would essentially be absent. The combination of the two modules therefore represents significant added value in terms of transport safety.

## 4. Results

A total of 242 individuals participated in the study, including 163 men and 79 women. The average age of the participants was 45.6 years (SD = 8.9). Based on clinical interviews and standardised questionnaires, the sample was divided into two groups: 81 people (33.5%) belonged to the depressed group, while 161 people (66.5%) belonged to the control group. There were no statistically significant differences between the two groups in terms of age (t(240) = 0.84; *p* = 0.401) or gender distribution (χ^2^(1) = 1.27; *p* = 0.260), so the groups can be considered comparable from a demographic point of view.

The distribution of the main pupillometry and eye movement characteristics was checked using the Shapiro–Wilk test; most of the data showed an approximately normal distribution, so parametric statistical methods were used in the analyses.

The performance of the iris-based identification module was evaluated separately for the 512-bit and 1024-bit encoding methods. In both configurations, the identification decision was based on the Hamming distance threshold. In the case of 512-bit encoding, the Genuine Acceptance Rate was 99.1%, while the False Acceptance Rate was 0.34% and the False Rejection Rate was 0.56%. The Equal Error Rate in this case was 0.45%.

The use of 1024-bit encoding further reduced the rate of unauthorised acceptances: the FAR decreased to 0.12%, while the GAR was 99.5%. The False Rejection Rate in this configuration increased to 0.62%, while the EER decreased to 0.37%. In both cases, the EER remained below 0.5%, indicating stable and reliable biometric identification performance and providing a suitable basis for further analysis based on individual reference data.

In the pupillometry analyses, we compared the participants’ responses to emotional stimuli with their individual resting baseline values. In response to news items with negative emotional content, the average pupil dilation in the control group was +18.4% (SD = 6.7%), while in the depressed group it was +27.9% (SD = 8.3%). The difference between the two groups was statistically significant (t(240) = 9.11; *p* < 0.001), and the effect size was considered large (Cohen d = 1.17). Based on the data, members of the depressed group showed a stronger or prolonged pupillary response to negative emotional stimuli, which was consistent across the entire sample.

Pupillometric responses to news with positive emotional content showed a different pattern. In the control group, the average pupil diameter increased by +6.2% (SD = 4.1%), while in the depressed group, the average change was −1.3% (SD = 5.0%), indicating a lack of pupil response or a slight constriction. The difference between the groups was also significant in this case (t(240) = 10.02; *p* < 0.001), with an effect size of d = 1.30. The reduced physiological response to positive emotional stimuli was consistently present in the depressed group and clearly distinguished the two groups.

In the analysis of eye movement characteristics, fixation time and saccade speed served as the main variables. The average fixation time measured during negative emotional stimuli was 280 ms (SD = 40 ms) in the control group and 345 ms (SD = 52 ms) in the depressed group. The difference was statistically significant (t(240) = 9.48; *p* < 0.001), and the effect size was d = 1.22.

The average saccade speed in the control group was 320°/s (SD = 45°/s), while in the depressed group it was 276°/s (SD = 51°/s). The difference was also significant in this case (t(240) = −7.01; *p* < 0.001), with an effect size in the medium-to-large range (d = 0.90). Based on the results, the gaze shifts of the depressed participants were slower, while their attention was fixed on a single stimulus for a longer period of time.

We evaluated the performance of a CNN–LSTM architecture depression detection model trained on pupillometry and eye movement time series data on a separate test sample. The accuracy of the model was 0.89. The sensitivity of depression detection was 0.87, while the specificity indicating the correct identification of non-depressed status was 0.90. The precision value was 0.84 and the F1 score was 0.85. The area under the ROC curve was 0.94, indicating excellent discriminatory power.

Based on the confusion matrix, the model incorrectly classified 16 control subjects as depressed, while failing to recognise depression in 11 cases. The effect of modifying the decision threshold was analysed separately. A probability threshold of 0.5 resulted in a balanced compromise between sensitivity and specificity. Lowering the threshold to 0.4 increased sensitivity (0.93) but decreased specificity (0.82), while raising the threshold to 0.6 resulted in decreased sensitivity (0.79) and increased specificity (0.94). The results show that the decision parameters of the model can be flexibly adjusted to the risk requirements of the application environment.

The results indicate that the integrated use of iris-based identification and pupillometry and eye movement-based depression detection reliably distinguishes between depressive and non-depressive patterns and provides a stable technical basis for a preventive, safety-critical transport system.

## 5. Discussion

The results of the study indicate that the integrated use of iris-based identification and pupillometry- and eye movement-based depression detection reliably distinguishes between depressive and non-depressive patterns. The consistently low Equal Error Rate values observed for both the 512-bit and 1024-bit iris encoding configurations demonstrate stable and reliable biometric identification performance, providing a secure individual reference framework for subsequent physiological analyses. This reliability is particularly important in safety-critical transport applications, where accurate identity verification and state monitoring must operate simultaneously and without interruption.

The pupillometry findings revealed clear group-level differences in responses to emotionally valenced stimuli. Participants in the depressed group showed significantly stronger and more prolonged pupil dilation in response to negative emotional content, while their responses to positive stimuli were markedly reduced or absent. These patterns suggest heightened sensitivity to negative information combined with blunted reactivity to positive stimuli, which is consistent with established affective processing characteristics of depression. The consistency of these effects across the sample supports the robustness of pupillometric measures as indicators of depression-related autonomic changes.

Eye movement analyses further reinforced these findings. Depressed participants exhibited longer fixation times and slower saccade speeds during exposure to negative emotional stimuli, indicating delayed attentional disengagement and prolonged focus on emotionally salient information. Together, the pupillometric and oculomotor results form a coherent physiological profile that differentiates depressive from non-depressive patterns and provides complementary information at both autonomic and attentional levels.

The CNN–LSTM-based depression detection model trained on pupillometry and eye movement time series data demonstrated high classification performance on an independent test sample. The observed accuracy, sensitivity, specificity, and excellent area under the ROC curve indicate strong discriminatory power. Analysis of different decision thresholds showed that the balance between sensitivity and specificity can be flexibly adjusted depending on application-specific risk requirements, which is particularly relevant for preventive systems in transport safety contexts. In this framework, the system is not intended to make clinical diagnoses but to provide early warning signals that may prompt further human evaluation or intervention.

Although these results are promising and suggest that the integration of artificial intelligence-based iris recognition and physiology-based depression detection may open new opportunities in transport safety, several methodological and practical limitations must be considered when interpreting the findings and assessing their applicability. One important limitation is that all measurements were conducted in a controlled laboratory environment with stable lighting conditions and minimal external distractions. While this setting enabled precise recording of pupillometry and eye movement data, it does not fully reflect real-life driving conditions. In real traffic environments, rapidly changing lighting, vibrations, noise, time pressure, and acute stress can all influence pupil dynamics and eye movement behaviour. The robustness of the proposed system under such conditions has not yet been validated, making further real-world testing essential.

Another methodological limitation relates to the characteristics of the participant sample. All participants were active, licenced drivers without known neurological or serious ophthalmological diseases. Conditions such as Parkinson’s disease and multiple sclerosis were mentioned not as target groups but as examples of disorders that can substantially affect pupillary reflexes and eye movements, thereby limiting the generalisability of the method. Since these conditions already restrict or exclude driving in most legal systems, the present study did not aim to identify or isolate them. Consequently, the model in its current form is applicable only to a healthy, active driving population.

The heterogeneous and dynamic nature of depression also represents a significant limitation. The study did not distinguish between different subtypes of depression, such as atypical, melancholic, or bipolar depression, which may exhibit distinct physiological patterns. As a result, model performance may vary across different forms of depression. In addition, long-term intra-individual variability was not examined, and it remains unclear how stable pupillometry and eye movement characteristics are within the same individual over extended periods.

A further limitation is that the physiological patterns identified by the model are not specific to depression. Temporary states such as fatigue, sleep deprivation, acute stress, hangovers, or the effects of certain medications can similarly influence pupil responses and eye movement dynamics. Although the use of individual baseline values reduces the impact of these confounding factors, it does not completely eliminate the possibility that transient conditions may produce depression-like physiological patterns. In its current form, the model cannot reliably distinguish clinical depression from short-term physiological or psychological states.

The use of emotionally valenced news stimuli for emotional elicitation also introduces limitations. Despite independent annotation of emotional content, individual interpretation, personal relevance, and current life circumstances can significantly influence emotional responses. Consequently, pupillometric reactions may be partly shaped by subjective factors, limiting complete standardisation of the stimuli.

From a technical perspective, the current system requires high-resolution imaging and relatively stable lighting conditions. In real-world applications, eyeglasses, contact lenses, makeup, eye fatigue or irritation, and vehicle-induced micro-vibrations may reduce measurement accuracy. Additionally, pharmacological factors such as antidepressants, stimulants, or sedatives can directly affect pupil function, yet these effects are not currently accounted for by the model.

Finally, it is essential to emphasise that although the system can identify physiological patterns associated with depression, it is not suitable for clinical diagnosis and does not replace medical or psychological assessment. Its sole purpose is to provide early warning of potential road safety risks, which may warrant further human decision-making or investigation. Ethical considerations, data protection, and the requirement for human oversight are therefore critical to ensure that automated assessments do not lead to irreversible consequences without expert review. Overall, the research represents an important first step toward the combined use of biometric identification and physiology-based depression detection in road safety applications. At the same time, the limitations discussed above highlight the need for further validation, real-world testing, and targeted methodological refinement before such systems can be widely deployed in practical driving environments.

## 6. Conclusions

The study presented a non-invasive and objective approach based on artificial intelligence for the detection of physiological patterns indicative of depression, integrating the analysis of pupillometric and eye movement-based signals with iris-based biometric identification. The central goal of the proposed framework is not to establish a clinical diagnosis, but to detect early risk conditions that could potentially compromise road and air transport safety. This approach emphasises the role of human factors in transport safety and offers a new perspective on monitoring mental state at the critical moment before driving.

Based on the results of the study, it can be concluded that the cognitive–affective distortions characteristic of depression consistently appear in the physiological responses of the eye. Participants showing depressive symptoms responded to negative emotional stimuli with increased pupil dilation, while a reduced or minimal response was observed in the case of positive stimuli. This pupillometric asymmetry was complemented by slower saccade speed and longer fixation times, which can be interpreted as behavioural manifestations of narrowed attention focus and reduced cognitive flexibility. These results are consistent with neurocognitive models of depression and support the notion that eye movements and pupillary reflexes are not merely peripheral phenomena, but sensitive indicators of neural information processing.

The presented CNN–LSTM-based deep learning model was able to capture complex, non-linear patterns in time-series pupillometry and eye movement data and achieved high classification performance in the study. This suggests that state-of-the-art artificial intelligence architectures may already be suitable for real-time processing tasks that are relevant in safety-critical environments. It is particularly important that the model is based on dynamic response patterns rather than static characteristics, which allows for more sensitive tracking of state changes.

One of the most important scientific and practical contributions of the research is the application of a longitudinal approach based on individual baseline values. Depression is a heterogeneous and time-varying condition whose physiological manifestations show significant inter- and intra-individual variability. Accordingly, approaches based on population averages or fixed thresholds carry a significant risk of misclassification. In contrast, the proposed system compares current measurements to the individual’s own previously recorded reference states, which significantly increases reliability and reduces the distorting effect of non-specific physiological changes such as fatigue, temporary stress or environmental influences.

The integration of iris-based biometric identification further reinforces this personalised approach. Identification not only performs an access control function, but also ensures that physiological data is always assigned to the same person, thus enabling the creation of a reference database that can be interpreted over the long term. This integration clearly distinguishes the presented framework from most current driver monitoring systems, which typically operate in a non-personalised manner and do not address individual patterns of mental states.

From a road safety perspective, the presented approach is preventive in nature. The system is not intended to automatically exclude or penalise drivers, but to provide early warning of potentially dangerous mental states. This approach is consistent with the design principles of modern safety-critical systems, which consider the support of human decision-making, rather than its replacement, to be the primary goal. In this context, the detection of depression is not a health assessment, but rather risk assessment information that may justify considering further steps, such as rest periods, secondary checks or alternative solutions.

However, the study also clearly points out the limitations of the method. The study was not conducted in a clinical population, and the determination of depressive status was based on self-administered questionnaires and structured interviews rather than psychiatric diagnosis. Accordingly, the results primarily relate to the physiological correlates of depressive symptoms and cannot be directly generalised for clinical diagnostic purposes. A further limitation is the individual relevance of the emotional stimulus, which, despite careful standardisation, may have influenced the intensity of the measured responses.

These factors underscore the importance of future research to validate the presented approach in larger samples, over longer periods of time, and in different contexts. Areas for further development include the integration of multimodal sensor data, such as combining pupillometry and eye movement characteristics with skin conductance, heart rate variability, or other autonomic nervous system signals. Such multimodal approaches are expected to further increase the robustness and specificity of the system.

Also of particular importance is the application of explainable artificial intelligence (XAI) methods, which can contribute to the transparency of decisions and facilitate the acceptance of the system from a legal and ethical perspective. The results of the study suggest that pupillometry and eye movement-based analysis supported by artificial intelligence is a viable and promising approach to identifying road safety risks associated with depression.

The individual baseline-based, biometrically identified approach opens up new possibilities for addressing human factors and can contribute to the development of prevention-focused road safety systems. With appropriate ethical, legal and technical safeguards in place, the framework presented here could play an important role in the future toolkit for decision support in safety-critical applications.

## Figures and Tables

**Table 1:** Comparison of methods for recognising depression source [4].

Method	Advantages	Disadvantages	Measurement Time	Required Tools
Clinical interview	Personalised	Subjective	High	Psychologist
Questionnaire assessment	Fast	Easy to manipulate	Low	paper/online
Pupillometry	Objective	Sensitive to lighting conditions	Medium	camera + light
Eye tracking	Rich physiological data	Expensive technology	Medium	eye tracker
AI-based model	Real-time, individual baseline	Requires a lot of data	Low	camera + AI

**Table 3:** Most common human factors in accidents (%) source [51].

Human Factor	Estimated Prevalence
Inattention	40–55
Fatigue	15–20
Cognitive load	10–15
Stress/emotional state	10–20
Alcohol/drugs	2–5

**Table 4:** ML models and input signals source [45].

ML Model	Input Signal	Typical Task
1D CNN	EDA, HRV	Stress detection
DNN	PPG, HR	Physiological state
SVM	Multimodal	Anxiety
Random Forest	EDA + HR	Emotional categories
HMM	EDA/HRV	Sleep stages

**Table 5:** Measurement parameters for the three good/bad news items source.

Parameter	Normal Reaction	Deviation Indicating Depression	AI Feature
Pupil dilation in response to bad news	female	excessive or delayed	pupil size
Pupil reaction to good news	female	decreases/no change	pupil reflex curve
Eye movement speed	stable	slows down/stalls	saccade speed
Fixation time	variable	too long	fixation length

## Data Availability

The data presented in this study are available on request from the corresponding author.

## References

[1] Ostacher M.J., Suppes T. (2018). Depression with mixed features in major depressive disorder: A new diagnosis or there all along?. J. Clin. Psychiatry.

[2] Miret M., Ayuso-Mateos J.L., Sanchez-Moreno J., Vieta E. (2013). Depressive disorders and suicide: Epidemiology, risk factors, and burden. Neurosci. Biobehav. Rev..

[3] American Psychiatric Association (2013). Diagnostic and Statistical Manual of Mental Disorders.

[4] Mohamed I., El-Wakad M., Abbas K., Aboamer M., Mohamed N.A.R. (2024). Pupil diameter and machine learning for depression detection: A comparative study with deep learning models. Appl. Comput. Sci..

[5] de Belen R.A., Pincham H., Hodge A., Silove N., Sowmya A., Bednarz T., Eapen V. (2023). Eye-tracking correlates of response to joint attention in preschool children with autism spectrum disorder. BMC Psychiatry.

[6] Levantini V., Muratori P., Inguaggiato E., Masi G., Milone A., Valente E., Tonacci A., Billeci L. (2020). EYES are the window to the mind: Eye-tracking technology as a novel approach to study clinical characteristics of ADHD. Psychiatry Res..

[7] Huang G., Li Y., Zhu H., Feng H., Shen X., Chen Z. (2023). Emotional stimulation processing characteristics in depression: Meta-analysis of eye tracking findings. Front. Psychol..

[8] Liu X.-C., Chen M., Ji Y.-J., Chen H.-B., Lin Y.-Q., Xiao Z., Guan Q.-Y., Ou W.-Q., Wang Y.-Y., Xiao Q.-L. (2025). Identifying depression with mixed features: The potential value of eye-tracking features. Front. Neurol..

[9] Tian C., Duan L., Fu C., He J., Dai J., Zhu G. (2022). Study on the Correlation Between Iris Characteristics and Schizophrenia. Neuropsychiatr. Dis. Treat..

[10] Youssef U.M., Raya Y.M., Sehlo M.G., Gado O.M., Hussien F.M., Gad A.A., Said M. (2025). Exploring the relationship between the density of the iris colour and bipolar disorder: A case-control study, Egypt. Ann. Gen. Psychiatry.

[11] Daugman J. How iris recognition works. Proceedings of the International Conference on Image Processing.

[12] Alkoot F.M., Box H.P.O. (2012). A review on advances in iris recognition methods. Int. J. Comput. Eng. Res..

[13] Ma L., Tan T., Wang Y., Zhang D. (2004). Local intensity variation analysis for iris recognition. Pattern Recognit..

[14] Daugman J.G. (1993). High confidence visual recognition of persons by a test of statistical independence. IEEE Trans. Pattern Anal. Mach. Intell..

[15] Bowyer K.W., Hollingsworth K., Flynn P.J. (2008). Image understanding for iris biometrics: A survey. Comput. Vis. Image Underst..

[16] Proença H., Alexandre L.A. (2010). Iris recognition: Analysis of the error rates regarding the accuracy of the segmentation stage. Image Vis. Comput..

[17] Arsalan M., Naqvi R.A., Kim D.S., Nguyen P.H., Owais M., Park K.R. (2018). IrisDenseNet: Robust Iris Segmentation Using Densely Connected Fully Convolutional Networks in the Images by Visible Light and Near-Infrared Light Camera Sensors. Sensors.

[18] Jain A.K., Ross A., Prabhakar S. (2004). An introduction to biometric recognition. IEEE Trans. Circuits Syst. Video Technol..

[19] Calvo R.A., D’Mello S. (2010). Affect Detection: An Interdisciplinary Review of Models, Methods, and Their Applications. IEEE Trans. Affect. Comput..

[20] D’Mello S.K., Kory J. (2015). A Review and Meta-Analysis of Multimodal Affect Detection Systems. ACM Comput. Surv..

[21] Siegle G.J., Granholm E., Ingram R.E., Matt G.E. (2001). Pupillary and reaction time measures of sustained processing of negative information in depression. Biol. Psychiatry.

[22] Granholm E., Asarnow R.F., Sarkin A.J., Dykes K.L. (1996). Pupillary Responses Index Cognitive Resource Limitations. Psychophysiology.

[23] Armstrong T., Olatunji B.O. (2012). Eye Tracking of Attention in the Affective Disorders: A Meta-Analytic Review and Synthesis. Clin. Psychol. Rev..

[24] Beatty J., Lucero-Wagoner B. (2000). The Pupillary System. Handbook of Psychophysiology.

[25] Woody M.L., Gibb B.E. (2015). Integrating NIMH Research Domain Criteria (RDoC) into Depression Research. Clin. Psychol. Rev..

[26] Stolicyn A., Steele J.D., Seriès P. (2020). Prediction of depression symptoms in individual subjects with face and eye movement tracking. Psychol. Med..

[27] Cembrano D.J.V. (2023). Heterogeneiety of Depression: A Scoping Review. Master’s Thesis.

[28] LeCun Y., Bengio Y., Hinton G. (2015). Deep Learning. Nature.

[29] Krizhevsky A., Sutskever I., Hinton G.E. (2017). ImageNet Classification with Deep Convolutional Neural Networks. Commun. ACM.

[30] Hochreiter S., Schmidhuber J. (1997). Long Short-Term Memory. Neural Comput..

[31] Soleymani M., Pantic M., Pun T. (2012). Multimodal Emotion Recognition in Response to Videos. IEEE Trans. Affect. Comput..

[32] Tzirakis P., Trigeorgis G., Nicolaou M.A., Schuller B.W., Zafeiriou S. (2017). End-to-End Multimodal Emotion Recognition Using Deep Neural Networks. IEEE J. Sel. Top. Signal Process..

[33] Samek W., Wiegand T., Müller K.-R. (2017). Explainable Artificial Intelligence: Understanding, Visualising and Interpreting Deep Learning Models. arXiv.

[34] Lednicka B., Kubacka M. (2022). Semi-Empirical Model of Remote-Sensing Reflectance for Chosen Areas of the Southern Baltic. Sensors.

[35] Wickens C.D., Hollands J.G., Banbury S., Parasuraman R. (2013). Engineering Psychology and Human Performance.

[36] Daugman J. (2004). How Iris Recognition Works. IEEE Trans. Circuits Syst. Video Technol..

[37] Siegle G.J., Thompson W., Carter C.S., Steinhauer S.R., Thase M.E. (2007). Increased Amygdala and Decreased Dorsolateral Prefrontal BOLD Responses in Unipolar Depression: Related and Independent Features. Biol. Psychiatry.

[38] Parasuraman R., Riley V. (1997). Humans and Automation: Use, Misuse, Disuse, Abuse. Hum. Factors J. Hum. Factors Ergon. Soc..

[39] Endsley M.R. (1995). Toward a Theory of Situation Awareness in Dynamic Systems. Hum. Factors J. Hum. Factors Ergon. Soc..

[40] Stanton N.A., Young M.S., Walker G.H. (2007). The Psychology of Driving Automation: A Discussion with Special Reference to Trust. Int. J. Veh. Des..

[41] Ouyang K., Cheng H.H. (2017). Safety management and formal safety assessment: A study of the Taiwan area. Aeronaut. Aerosp. Open Access J..

[42] Montesinos V., Dell’Agnola F., Arza A., Aminifar A., Atienza D. Multi-modal acute stress recognition using off-the-shelf wearable devices. Proceedings of the 41st Annual International Conference of the IEEE Engineering in Medicine and Biology Society (EMBC).

[43] Padil H., Said M.N., Azizan A. (2018). The contributions of human factors on human error in Malaysia aviation maintenance industries. IOP Conf. Ser. Mater. Sci. Eng..

[44] Ancillon L., Elgendi M., Menon C. (2022). Machine learning for anxiety detection using biosignals: A review. Diagnostics.

[45] Hazer-Rau D., Zhang L., Traue H.C. (2020). A workflow for affective computing and stress recognition from biosignals. Eng. Proc..

[46] Saleh A.Y., Xian L.K. (2021). Stress classification using deep learning with 1d convolutional neural networks. Knowl. Eng. Data Sci..

[47] Kim A.Y., Jang E.H., Kim S., Choi K.W., Jeon H.J., Yu H.Y., Byun S. (2018). Automatic detection of major depressive disorder using electrodermal activity. Sci. Rep..

[48] Can Y.S., Gokay D., Kılıç D.R., Ekiz D., Chalabianloo N., Ersoy C. (2020). How laboratory experiments can be exploited for monitoring stress in the wild: A bridge between laboratory and daily life. Sensors.

[49] Hazer-Rau D., Meudt S., Daucher A., Spohrs J., Hoffmann H., Schwenker F., Traue H.C. (2020). The uulmmac database—A multimodal affective corpus for affective computing in human-computer interaction. Sensors.

[50] Kim D., Min J., Ko S.H. (2023). Recent developments and future directions of wearable skin biosignal sensors. Adv. Sens. Res..

[51] Shishido E., Ogawa S., Miyata S., Yamamoto M., Inada T., Ozaki N. (2019). Application of eye trackers for understanding mental disorders: Cases for schizophrenia and autism spectrum disorder. Neuropsychopharmacol. Rep..

[52] Piccini J., August E., Óskarsdóttir M., Arnardóttir E.S. (2023). Using the electrodermal activity signal and machine learning for diagnosing sleep. Front. Sleep.

[53] Mendez C., Kaykayoglu C.A., Bähler T., Künzler J., Lizoain A., Rothenbühler M., Schmidt M.H., Laimer M., Witthauer L. (2025). Toward detection of nocturnal hypoglycaemia in people with diabetes using consumer-grade smartwatches and a machine learning approach. J. Diabetes Sci. Technol..

[54] Li R., Liu Z. (2020). Stress detection using deep neural networks. BMC Med Inform. Decis. Mak..

[55] Jang E.H., Choi K.W., Kim A.Y., Yu H.Y., Jeon H.J., Byun S. (2022). Automated detection of panic disorder based on multimodal physiological signals using machine learning. ETRI J..

[56] Vargas E.P., Philip J., Carrasco-Ribelles L.A., Giglioli I.A.C., Valenza G., Marín-Morales J., Raya M.A. (2023). The neurophysiological basis of leadership: A machine learning approach. Manag. Decis..

[57] Wang L., Gao S., Zhang N. (2025). A psychophysiological model based on machine learning algorithms for evaluating commercial airline pilots’ mental workload in flight-simulation context. Hum. Factors Ergon. Manuf. Serv. Ind..

[58] Byun S., Kim A.Y., Jang E.H., Kim S., Choi K.W., Yu H.Y., Jeon H.J. (2019). Entropy analysis of heart rate variability and its application to recognise major depressive disorder: A pilot study. Technol. Health Care.

[59] Maritsch M., Föll S., Lehmann V., Styger N., Bérubé C., Kraus M., Feuerriegel S., Kowatsch T., Züger T., Fleisch E. (2023). Smartwatches for non-invasive hypoglycaemia detection during cognitive and psychomotor stress. Diabetes Obes. Metab..

[60] Čertický M., Čertický M., Sinčák P., Magyar G., Vaščák J., Cavallo F. (2019). Psychophysiological indicators for modelling user experience in interactive digital entertainment. Sensors.

[61] Blackmore K.L., Smith S.P., Bailey J.D., Krynski B. (2024). Integrating biofeedback and artificial intelligence into extended reality training scenarios: A systematic literature review. Simul. Gaming.

[62] Cowley B., Kosunen I., Lankoski P., Kivikangas J.M., Järvelä S., Ekman I., Kemppainen J., Ravaja N. (2014). Experience assessment and design in the analysis of gameplay. Simul. Gaming.

